# Association of Ginseng Consumption With All-cause and Cause-specific Mortality: Shanghai Women’s Health Study

**DOI:** 10.2188/jea.JE20210393

**Published:** 2022-10-05

**Authors:** Pranoti Pradhan, Wanqing Wen, Hui Cai, Yu-Tang Gao, Gong Yang, Xiao-ou Shu, Wei Zheng

**Affiliations:** 1 Division of Epidemiology, Department of Medicine, Vanderbilt Epidemiology Center, Vanderbilt-Ingram Cancer Center, Vanderbilt University Medical Center, Nashville, Tennessee, USA; 2 Department of Epidemiology, Shanghai Cancer Institute, Shanghai, China

**Keywords:** ginseng, mortality, epidemiology, cardiovascular diseases, alternative medicine

## Abstract

**Background:**

Ginseng, an herbal remedy, has been commonly used in Asian countries to promote longevity and health for over 2,000 years. However, the association of ginseng consumption with all-cause and cause-specific mortality is still unclear. We analyzed the association of total and major cause-specific mortality (cardiovascular disease [CVD], cancer, and other death) with consumption of ginseng (primarily American and white ginseng).

**Methods:**

This study included 56,183 female participants with an average follow-up of 14.7 years in the Shanghai Women’s Health Study, an ongoing prospective cohort study. Data were assessed via an in-person interview conducted at baseline recruitment. Cox proportional hazards models were used to estimate hazard ratios (HRs) and 95% confidence intervals (CIs) for ginseng-mortality associations after adjusting for confounders.

**Results:**

Compared with those who never used ginseng, regular ginseng use was associated with significantly reduced all-cause mortality (HR 0.92; 95% CI, 0.87–0.98). This inverse association was seen primarily among those who consumed ginseng for perceived general health benefit (HR 0.90; 95% CI, 0.85–0.96). A significant dose-response association was observed between duration of ginseng use and total mortality (HR 0.85, for using ≥6 years vs never use; *P* for trend <0.001), CVD mortality (HR 0.83; *P* for trend = 0.019), and other-cause mortality (HR 0.76; *P* for trend = 0.001). However, no dose-response association was observed between amount of ginseng consumption and mortality outcomes.

**Conclusion:**

Regular ginseng consumption, particularly over a long duration, was associated with decreased risk of all causes of death, death due to CVD, and death due to certain other diseases.

## INTRODUCTION

Over the past decades, there has been an increase in the use of alternative and complementary medical approaches, especially herbal medicines, to help alleviate disease symptoms and improve health.^1^^–^^5^ Ginseng, which is one of these herbal remedies, has been commonly used by Chinese to promote longevity and health for over 2,000 years.^4^ Throughout the years, ginseng has also been gaining popularity in other Asian and Western countries. *Panax ginseng* C.A. Meyer (Asian ginseng), known as white ginseng, and *Panax quinquefolius* L. (American ginseng) are the two most commonly used ginsengs in Asian and Western countries, respectively.^6^

Previous studies have shown potential benefits of ginseng on improving immune function and cardiovascular system responses and affecting sex hormone levels.^7^^–^^9^ Data from in vivo experiments with animal models has also suggested that ginseng and its constitutes, ginsenosides, have anti-inflammatory, anti-oxidative, and anti-diabetic properties.^8^^–^^10^ In addition, ginseng has been shown to potentially improve metabolism and cognitive development and decrease cell stress.^11^^,^^12^

Epidemiologic studies on the association between ginseng consumption and risk of diseases have been limited. Several studies have evaluated the association of ginseng consumption with risk of cancer^13^^,^^14^ and cardiovascular disease (CVD).^15^^–^^17^ Results from these studies, however, have been inconsistent. To our knowledge, only one small cohort study, which included less than 6,300 participants, has investigated the association between ginseng consumption and all-cause and cause-specific mortality.^15^ The study reported some reduction in mortality risk in association with regular ginseng consumption, but the results were inconsistent by sex. We evaluated the association of ginseng use with total and cause-specific mortality (all-cause death, CVD, cancer, and other death) in a population-based cohort study of 56,183 female participants with nearly 15 years of follow-up.

## METHODS

### Study population

Data used in this study were from a population-based cohort study, the Shanghai Women’s Health Study (SWHS). The details of this study have been published elsewhere.^18^ In brief, the SWHS recruited 74,940 women between the ages of 40–70 years old living in urban communities in Shanghai.^18^ In-person interviews were performed from 1996 to 2000 at the cohort baseline survey to obtain information on demographic characteristics, lifestyle habits, personal and familial disease history, and other known and suspected risk factors for chronic diseases, with an overall response rate of 92.7%.^18^^,^^19^ Anthropometric measurements were obtained at baseline by trained interviewers. Written informed consent was obtained from all study participants. The study was approved by the institutional review boards of all participating institutions and the work is supported by the National Institutes of Health (grant number UM1 CA182910). Information on the use of ginseng (ginseng root) or a ginseng product was obtained through in-person interviews. Participants were asked if they had regularly consumed ginseng (at least five times a year as defined in the questionnaire) within the past 3 years.^18^ For those participants who reported regularly using ginseng, information was ascertained. This included the type of ginseng used (white or red Asian ginseng, American ginseng, and ginseng products (eg, root extract, powder, tablet, or capsule), the duration (years) of use, the amount (grams/years) of use, and the reason for use.^18^ All interviewers received a 2-week rigorous training, and only qualified interviewers were certified to conduct surveys. To further improve the quality of survey data, all in-person interviews were tape-recorded, and more than 5% of recorded interviews were evaluated by the study quality control team.

Information on vital status and causes of deaths was obtained through a combination of in-person follow-up surveys and record-linkage with the database of the Shanghai Cancer Registry and the Shanghai Vital Statistic Registry. Five in-person follow-up surveys were conducted from 2000 to 2018, with response rates of 99.76%, 98.69%, 95.03%, 92.50%, and 91.01% for the first to the fifth survey, respectively. These in-person follow-ups, conducted every 2 to 4 years for all living cohort members, were to update exposure data and collect information on health status, diseases diagnosis, and new exposure information. Ascertainment of mortality outcomes is virtually complete via the linkage with registry data because of the extremely low outmigration rate.

### Statistical analysis

**Figure 1.**
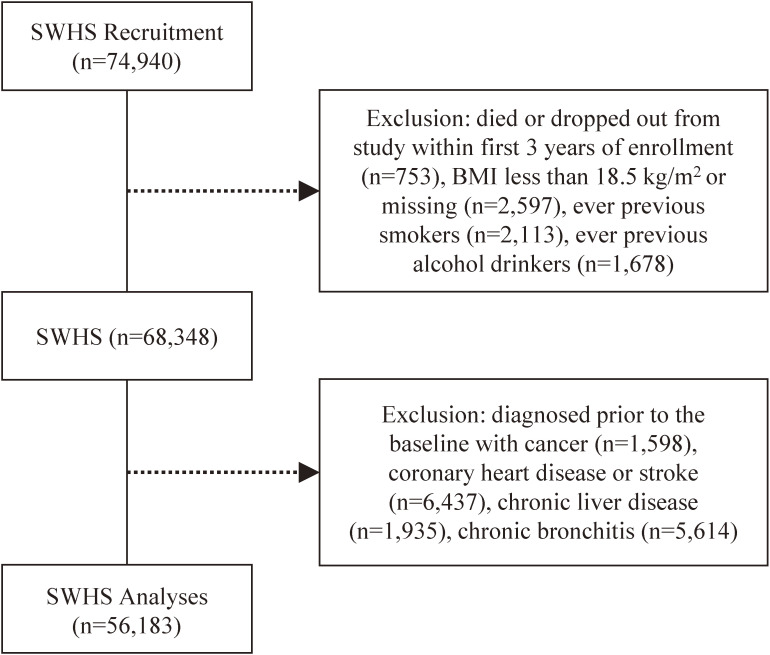
Flow diagram of the study population selection from Shanghai Women’s Health Study (SWHS) cohort. The results are not mutually exclusive.

The primary endpoints for this analysis were all-cause and major cause specific mortality. The International Classification of Diseases (ICD) Ninth Revision codes were used to classify the causes of death. Deaths with ICD codes 140–208 were classified as cancer, and deaths with ICD codes 390–459 were classified as CVD mortality. We also excluded those who had history of cancer, coronary heart disease, or stroke at baseline; had chronic liver disease or chronic bronchitis; or died within the first 3 years after study enrollment to minimize possible influence of reverse causality in our results. Person-years cumulated for the first 3 years were also excluded in the analysis. Very few cohort members had missing body mass index (BMI; 0.05%), BMI less than 18.5 kg/m^2^ (4.58%), or were previous cigarette smokers (3.76%) or alcohol drinkers (2.99%), so these individuals were also excluded from the current analysis to remove their potential influence to confound the results. After excluding those participants, 56,183 women remained for the current analysis (Figure 1). The average follow-up time for this cohort was 14.7 years.

We compared distributions of selected baseline characteristics of study participants by ginseng consumption status with the *t*-test for continuous variables and the chi-square test for categorical variables. The hazard ratios (HRs) and 95% confidence intervals (CIs) for the association of ginseng consumption with subsequent overall or cause-specific mortalities were analyzed using Cox proportional hazards models. Person-years were counted from 1996 up to December 31, 2016 or censoring on the date of death, whichever came first. Our analyses were primarily focused on white and American ginseng, as the vast majority of ginseng used in the population were these two types. Total amount of ginseng consumption for both white and American ginseng combined was categorized into three groups, which are high (≥750 g per year), medium (375–749.99 g per year), and low use (<375 g per year). The duration of ginseng usage was categorized as short (<3 years), medium (3–5.99 years), and long-term (≥6 years) use. Reasons for consumption of ginseng were categorized as either consumed for perceived general benefit for enhancement of health, for perceived specific health benefit against existing/prevalent illness, or for other reasons.

The Cox models were adjusted for potential confounders determined based on a priori knowledge, which included age at baseline survey, waste-to-hip ratio (WHR), BMI (categorized 18.5–19.9, 20.0–24.9, 25.0–29.9, ≥30.0 kg/m^2^), education (elementary school or less, middle school, high school graduate, some college or higher); household income (low: <10,000 yuan; middle: 10,000–30,000 yuan; high: >30,000 yuan); marital status (currently married, single/separated/divorced/widowed); menopause status (yes, no); exercise (continuous metabolic equivalent of tasks [MET]s); vitamin consumption (yes, no); calcium consumption (yes, no); diet (categorical quartiles, healthy dietary score); and ever diagnosed with hypertension (yes, no) or diabetes (yes, no).^13^^–^^16^^,^^18^^–^^20^ The healthy dietary score was calculated based on 8 food groups: fruit, vegetables (excluding potatoes), dairy, fish and seafood, nuts and legumes, refined grains, red meat, and processed meat.^20^^,^^21^ The methods are described elsewhere, but, in brief, the score was based on energy adjusted and sex-specific quintiles.^20^^,^^21^ The first five food groups were given ascending values (1 to 5) and the last three groups were given descending values (5 to 1).^20^^,^^21^ The healthy dietary score was the sum of those values, and a higher score corresponded with a better diet.^20^^,^^21^ Within these covariates, 8 out of 56,183 individuals had missing data for education (0.01%) and 11 out of 56,183 individuals had missing data for income (0.02%). Since the missing rates were very low, individuals with missing values for descriptive statistics were included in the analysis but were excluded from the multivariate modeling.

Lastly, *P* values for trend tests were derived from the regression model by treating the ordered ginseng consumption amount or duration as a continuous variable. Statistical analyses were performed using STATA IC (Version 16.0.801; Stata Corp., College Station, TX, USA) and R (Version: 1.2.1335, R Foundation for Statistical Computing, Vienna, Austria. All statistical tests were two-sided, and a *P* value of less than 0.05 was considered statistically significant.^22^

## RESULTS

Selected demographic factors and major risk factors for mortality of the participants at baseline are presented in Table 1. At baseline, 14,270 (25.4%) women reported using ginseng regularly (regular users). In this study population, the all-cause mortality was 8.82 per 1,000 person-years, mortality due to CVD was 3.06 per 1,000 person-years, mortality due to cancer was 3.52 per 1,000 person-years, and mortality due to other causes was 2.24 per 1,000 person-years. Compared to nonusers, regular ginseng users were on average older, more educated, had a higher income, and exercised more regularly. However, regular ginseng users also had higher frequencies of hypertension and diabetes. Furthermore, American ginseng and white ginseng were the two most commonly used types of ginseng, accounting for approximately 97.1% of total ginseng use.

**Table 1.** Selected baseline characteristics of study participants by ginseng consumption status, Shanghai Women’s Health Study, 1996–2016

Characteristics	Regular ginseng use		

	No	Yes^b,c^	*P*-value
Number of cohort members	41,913	14,270	
Age, years^a^	50.4 (8.3)	54.1 (9.1)	<0.001
Waist-to-hip ratio^a^	0.808 (0.05)	0.811 (0.05)	<0.001
Body mass index, kg/m^2 a^	24.1 (3.2)	24.0 (3.1)	0.456
Education, %			<0.001
Never have formal education	7.7	11.8	
Elementary school or less	8.2	13.0	
Middle school	42.2	31.4	
High school graduate	29.3	27.6	
Some college and higher	12.6	16.1	
Household income, %			<0.001
Low	15.4	14.2	
Middle	75.3	73.9	
High	9.3	11.9	
Currently married, %	90.8	87.4	<0.001
Ever diagnosed with hypertension, %	18.8	24.0	<0.001
Ever diagnosed with diabetes, %	2.9	5.0	<0.001
Ever consumed vitamin supplements, %	5.3	10.6	<0.001
Ever consumed calcium supplements, %	14.8	24.1	<0.001
LTPA daily activity, %	22.7	36.1	<0.001

LTPA, leisure-time physical activity; SD, standard deviation.

^a^Mean (SD).

^b^Median (interquartile range [IQR]) for duration of ginseng consumption: 4 (IQR, 2–9 years).

^c^Median (IQR) for amount of ginseng consumption: 100 (IQR, 50–150 grams).

Table 2 presents the associations between ginseng use (ever vs never) and mortality outcomes in all participants and by reasons of ginseng use. Ginseng use was found to be significantly associated with decreased risk of all causes of death among all participants after adjustment for covariates (HR 0.92; 95% CI, 0.87–0.98). This association held for cause-specific mortality, although it was not statistically significant for deaths due to CVD and cancers. Stratified analysis by reasons of ginseng use, the association of consumption of ginseng and decreased risk of deaths was more evident among those who consumed ginseng for the perceived general benefit for enhancement of health (HR 0.90; 95% CI, 0.85–0.96 for all causes of deaths, HR 0.91; 95% CI, 0.82–0.99 for deaths due to CVD, and HR 0.79; 95% CI, 0.69–0.90 for deaths due to unspecified causes). Similar results were found when we restricted analyses to American ginseng and white ginseng. No significant association, however, was found among cohort members who consumed ginsengs for perceived specific health benefits against existing diseases, likely due to possible influence of reserve causation (eTable 1). Therefore, all subsequent analyses were performed to evaluate the association of mortality with the use of American ginseng and white ginseng among individuals who consumed ginseng for perceived general benefit for enhancement of health, which was the reason for the majority of participants who consumed ginseng. After this stratification, there were 12,922 women who consumed American ginseng and white ginseng for this reason.

**Table 2.** HRs^a^ and 95% CIs of mortality associated with ginseng use, Shanghai Women’s Health Study

Ginseng Consumption	Adjusted HR (95% CI)^a^		Adjusted HR (95% CI)^a^		Adjusted HR (95% CI)^a^		Adjusted HR (95% CI)^a^	
	among all subjects^b^		among consumers for general health benefits^c^		among consumers for specific health benefits against existing illness^d^		among consumers for other reasons^e^	
			
	Deaths (*N*)	Total Users	Deaths (*N*)	Total Users	Deaths (*N*)	Total Users	Deaths (*N*)	Total Users
***All causes***								
Never	3,427	1.00	3,427	1.00	3,427	1.00	3,427	1.00
Ever	1,681	0.92 (0.87–0.98)	1,533	0.90 (0.85–0.96)	186	1.09 (0.94–1.27)	50	0.85 (0.64–1.12)
American/White ginseng	1,578	0.92 (0.87–0.98)	1,437	0.90 (0.85–0.96)	138	1.24 (1.03–1.48)	7	0.88 (0.43–1.79)
***Cardiovascular diseases***								
Never	1,049	1.00	1,049	1.00	1,049	1.00	1,049	1.00
Ever	575	0.92 (0.83–1.02)	531	0.91 (0.82–0.99)	54	0.93 (0.70–1.24)	16	0.88 (0.53–1.45)
American/White ginseng	544	0.93 (0.84–1.04)	504	0.93 (0.83–1.04)	40	1.04 (0.74–1.45)	4	1.96 (0.76–5.06)
***Cancer***								
Never	1,551	1.00	1,551	1.00	1,551	1.00	1,551	1.00
Ever	703	0.96 (0.88–1.05)	657	0.96 (0.87–1.05)	67	1.04 (0.81–1.34)	25	0.98 (0.66–1.46)
American/White ginseng	655	0.95 (0.87–1.05)	611	0.95 (0.86–1.04)	42	1.03 (0.75–1.41)	2	0.49 (0.12–1.95)
***Other disease causes***								
Never	827	1.00	827	1.00	827	1.00	827	1.00
Ever	403	0.85 (0.75–0.96)	345	0.79 (0.69–0.90)	65	1.31 (1.01–1.69)	9	0.59 (0.32–1.11)
American/White ginseng	379	0.86 (0.76–0.97)	322	0.79 (0.69–0.90)	56	1.69 (1.27–2.24)	1	0.57 (0.08–4.15)

CI, confidence interval, HR, hazard ratio.

^a^Adjustment: age, income, marriage status, education, menopause status, comorbidity (hypertension, diabetes), exercise, waist-to-hip ratio, body mass index, diet, vitamin and calcium supplements.

^b^Of the 14,270 participants who ever regularly consumed ginseng, 13,830 consumed American/White ginseng.

^c^Of the 13,308 participants who ever regularly consumed ginseng for general health benefits, 12,922 consumed American/White ginseng.

^d^Of the 869 participants who ever regularly consumed ginseng specific health benefits against existing illness, 821 consumed American/White ginseng.

^e^Of the 107 participants who ever regularly consumed ginseng for other reason, 101 consumed American/White ginseng.

In Table 3, we categorized the use of American ginseng and white ginseng to three durations (<3 years, 3–5.99 years, and ≥6 years). There were 538 women with missing data on their duration of American ginseng and white ginseng consumption and thus were excluded from the analysis. We found that the longer use was associated with lower risk when comparing with never users, in a linear dose-response manner, for all causes of death (*P* for trend <0.001), and death due to CVD (*P* for trend = 0.019) and other causes of death (*P* for trend = 0.001). However, no dose-response relation was found between duration of ginseng use and cancer mortality (*P* for trend = 0.100).

**Table 3.** HR^a^ and 95% CI of mortality associated with duration of consuming either American or White ginseng among individuals who consumed ginseng for perceived general benefit for enhancement of health, Shanghai Women’s Health Study

Cause of deaths		Duration of ginseng use, years			

	Never Use (*N* = 41,431)	<3 (*N* = 3,089)	3–5.99 (*N* = 3,976)	≥6 (*N* = 5,319)	*P* for trend
***All causes***					
Deaths (*N*)	3,427	318	445	611	
HR (95% CI)	1.00	0.98 (0.87–1.10)	0.90 (0.81–0.99)	0.85 (0.78–0.92)	<0.001
***Cardiovascular diseases***					
Deaths (*N*)	1,049	113	162	208	
HR (95% CI)	1.00	1.07 (0.88–1.30)	0.94 (0.79–1.11)	0.83 (0.71–0.96)	0.019
***Cancer***					
Deaths (*N*)	1,551	141	178	264	
HR (95% CI)	1.00	1.05 (0.88–1.25)	0.90 (0.77–1.06)	0.91 (0.80–1.04)	0.100
***Other causes***					
Deaths (*N*)	827	64	105	139	
HR (95% CI)	1.00	0.77 (0.59–0.99)	0.82 (0.67–1.00)	0.76 (0.63–0.91)	0.001

CI, confidence interval, HR, hazard ratio.

^a^Models adjusted for age, income, marriage status, education, menopause status, comorbidity (hypertension, diabetes), exercise, waist-to-hip ratio, body mass index, diet, vitamin and calcium supplements.

Participants (*N* = 538) with missing data on duration of ginseng use were excluded.

Table 4 presents the association of deaths with the amount of American ginseng and white ginseng consumption. A low amount (<375 grams/year) of American ginseng and white ginseng consumption was associated with significantly decreased risk of all causes of death (HR 0.86; 95% CI, 0.78–0.94), deaths due to CVD (HR 0.82; 95% CI, 0.69–0.97), and other cause of death (HR 0.64; 95% CI, 0.51–0.80) when compared with never users. Although no clear dose-response association was found between the amount of ginseng consumption and mortality outcomes, trend tests for a linear association were statistically significant for all causes of death (*P* for trend = 0.017) and other causes of death (*P* for trend = 0.005).

**Table 4.** HRs^a^ and 95% CIs of mortality associated with amount of consuming either American or White ginseng among individuals who consumed ginseng for perceived general benefit for enhancement of health, Shanghai Women’s Health Study

Cause of deaths	Never Use (*N* = 41,431)	Amount of ginseng use, grams/year			

		<375 (*N* = 4,270)	375–749.99 (*N* = 5,095)	≥750 (*N* = 3,557)	*P*-trend
***All causes***					
Deaths (*N*)	3,427	466	603	368	0.017
HR (95% CI)	1.00	0.86 (0.78–0.94)	0.93 (0.85–1.01)	0.92 (0.82–1.03)	
***Cardiovascular diseases***					
Deaths (*N*)	1,049	160	197	147	0.989
HR (95% CI)	1.00	0.82 (0.69–0.97)	0.89 (0.76–1.03)	1.15 (0.96–1.38)	
***Cancer***					
Deaths (*N*)	1,551	216	247	148	0.110
HR (95% CI)	1.00	1.01 (0.88–1.17)	0.95 (0.83–1.09)	0.87 (0.72–1.02)	
***Other causes***					
Deaths (*N*)	827	90	159	73	0.005
HR (95% CI)	1.00	0.64 (0.51–0.80)	0.95 (0.80–1.12)	0.71 (0.55–0.91)	

CI, confidence interval, HR, hazard ratio.

^a^Adjustment: age, income, marriage status, education, menopause status, comorbidity (hypertension, diabetes), exercise, waist-to-hip ratio, body mass index, diet, vitamin and calcium supplements.

All HRs and 95% CI are calculated compared with never users.

We further analyzed the joint association of the amount and duration of ginseng use for either American or White ginseng with mortality (Table 5). The inverse association of ginseng use was most evident among long-term users (≥3 years) who took a low amount (<375 grams/year), who had lower risk of all causes of deaths (HR 0.83; 95% CI, 0.73–0.93), death due to CVD (HR 0.79; 95% CI, 0.64–0.97), and other cause of death (HR 0.58; 95% CI, 0.44–0.77) than never users.

**Table 5.** The joint associations^a^ of the amount and duration of consuming either American or White ginseng with mortality among individuals who consumed ginseng for perceived general benefit for enhancement of health, Shanghai Women’s Health Study

Cause of deaths		Amount of ginseng use, grams/year			

		<375 g (*N* = 4,270)	375–749.99 g (*N* = 5,095)	≥750 g (*N* = 3,557)	*P*-trend
***All causes***					
*<3 years*	Deaths (*N*)	160	156	64	0.584
	HR (95% CI)	0.91 (0.78–1.07)	1.11 (0.95–1.31)	1.02 (0.79–1.30)	
*≥3 years*	Deaths (*N*)	306	446	304	0.001
	HR (95% CI)	0.83 (0.73–0.93)	0.87 (0.79–0.96)	0.90 (0.80–1.01)	
***Cardiovascular diseases***					
*<3 years*	Deaths (*N*)	53	59	22	0.129
	HR (95% CI)	0.88 (0.67–1.17)	1.31 (0.99–1.71)	1.21 (0.79–1.85)	
*≥3 years*	Deaths (*N*)	107	138	125	0.400
	HR (95% CI)	0.79 (0.64–0.97)	0.78 (0.65–0.93)	1.14 (0.94–1.38)	
***Cancer***					
*<3 years*	Deaths (*N*)	73	65	30	0.388
	HR (95% CI)	1.03 (0.82–1.31)	1.11 (0.87–1.43)	1.07 (0.74–1.54)	
*≥3 years*	Deaths (*N*)	143	181	118	0.023
	HR (95% CI)	1.01 (0.85–1.19)	0.89 (0.76–1.04)	0.82 (0.68–0.99)	
***Other causes***					
*<3 years*	Deaths (*N*)	34	32	12	0.089
	HR (95% CI)	0.76 (0.54–1.07)	0.88 (0.61–1.25)	0.70 (0.38–1.29)	
*≥3 years*	Deaths (*N*)	56	127	61	0.009
	HR (95% CI)	0.58 (0.44–0.77)	0.96 (0.80–1.16)	0.71 (0.54–0.93)	

CI, confidence interval, HR, hazard ratio.

^a^Adjustment: age, income, marriage status, education, menopause status, comorbidity (hypertension, diabetes), exercise, waist-to-hip ratio, body mass index, diet, vitamin and calcium supplements.

All HRs and 95% CI are calculated compared with never users.

Of the participants who regularly consumed American/White ginseng, 3,832 participants consumed it for less than 3 years.

Of the participants who regularly consumed American/White ginseng, 9,957 participants consumed it for 3 or more years.

## DISCUSSION

In this large prospective cohort study with a long-term follow-up, we found that ginseng use, particularly long-term ginseng use for improvement of general health, was associated with decreased risk of all causes of deaths, death due to CVD, and other causes of death. Our results suggested that regular ginseng consumption may confer some health benefits in reducing mortality.

Our results are supported, in part, by a previous small study conducted in a Korean population, in which a decrease in all-cause mortality for male ginseng users compared to non-users was observed (HR 0.90; 95% CI, 0.81–0.99).^15^ That study also reported a decreased cancer-specific mortality (HR 0.61; 95% CI, 0.32–1.14) in female regular ginseng users. Yun et al reported a suggestive dose-response relationship between increased ginseng intake and decreased cancer risk.^23^ However, our study did not find any significant association between ginseng use and cancer-specific mortality. The reason for the inconsistency about cancer outcomes between our analysis and the previous study results are unclear, and further studies are needed.

There are several lines of evidence to support the inverse association of ginseng use with CVD mortality observed in our study. Ginseng has been shown to improve arterial functions and facilitation of vasorelaxation.^24^^,^^25^ Specifically, Rg3 ginsenoside has been shown to have ACE activity inhibition and anti-inflammatory potential in in vitro experiments.^26^ In vivo studies also show that ginseng can lead to an improved lipid profile and a decreased risk of atherosclerosis by reducing plasma levels of cholesterol, free fatty acids, and triglycerides.^24^ A study by Kim also shows that ginseng intake could lead to decreased antithrombotic effects due to its antiplatelet activity, suggesting that consumption may be beneficial for those with elevated risk of CVD.^16^ These findings support our results, suggesting that ginseng intake could have potential beneficial effects in reducing risk of death due to CVD.

Ginseng may have health benefits beyond the cardiovascular system. Lü et al performed an in vivo study on mice demonstrating that ginseng helped extend lifespan, increased resistance to starvation stress, and also prevented weight gain.^8^ Ginseng enhanced the organism’s metabolism in favor of increasing lifespan and also portrayed anti-inflammatory, anti-oxidative, and anti-diabetic properties.^8^ A similar study by Szczuka et al expanded on these results, showing that ginsenosides could improve nervous system activity, cognitive function, and decrease hypertrophy.^9^ Specifically, the study team reported neuroprotective effects against neuronal damage resulting from ischemic stroke in animals, increased insulin sensitivity and inhibition of adipose tissue formation, and antimicrobial potential against several pathogenic strains of bacteria.^9^ These results from previous studies support our findings because increased biological activity and health benefits can help decrease all-cause mortality.

In this study, we were able to analyze the association of ginseng use with all causes of death and cause-specific death by the reasons of its use. We found that benefit of ginseng consumption primarily manifested among those who consumed ginseng for perceived general benefit for enhancement of health. However, the survival was not improved among those who consumed ginseng for perceived specific health benefit against existing or prevalent illness, suggesting that the therapeutic effects of ginseng use for particular diseases may be limited. In our study, we found that ginseng users were more likely to have a high income, a better education, and more existing comorbidity conditions, and they exercised more regularly than non-ginseng users. It is possible that high socio-economic status, healthy lifestyles, and poor health among ginseng users may have confounded the association. In our analysis, the associations of mortality with ginseng use held after fully adjusting for socioeconomic status and known lifestyle and disease risk factors for mortality.

Our analysis did not find a significant association between a larger amount of ginseng consumption and all-cause or cause-specific mortality. Previous literature reported that lower doses of ginseng has the same effects as larger doses of ginseng on cognitive function and conditions related to diabetes.^27^^,^^28^ However, these previous studies show that the duration of ginseng consumption may be more important than the amount of ginseng consumption, which is similar to our findings.^27^^,^^28^

Other noticeable strengths of our study include a prospective cohort design with a large sample size, detailed assessments of ginseng use history, and a high participation rate. All of these factors allowed us to perform a detailed analysis of the association of various levels and reasons of ginseng consumption with all-cause and cause-specific mortality. The study also has a high follow-up rate, enhancing the validity of the study findings. Additionally, the study includes a comprehensive assessment of demographic, lifestyle, and major disease risk factors conducted at the baseline survey, so we were able to control for a wide range of potential confounders. However, a major limitation of this study is that ginseng consumption was self-reported, which could introduce potential misclassification errors, particularly regarding the amount of ginseng consumption. These errors should be mostly non-differential due to the prospective design, leading to attenuation of the true association between the exposure and outcomes. To alleviate bias from these errors, we had the participants report ginseng consumption by not only total ginseng but also by individual ginseng type, amount, and the duration over years. Ginsengs are consumed as supplements, not as foods. Therefore, like most other dietary validation studies, we did not include ginsengs and other supplements in our dietary validation study.^29^ Therefore, we cannot provide any specific data regarding the accuracy of ginseng intake data. However, similar to tea drinking, it should be easier (and thus, more accurate) to measure ginseng consumption than dietary intakes. Using a method similar to the one used to assess ginseng intake, we obtained tea drinking information in this cohort study and showed that tea drinking was associated with a reduced risk of several cancers.^29^^,^^30^ Also, using a similar method, we obtained ginseng intakes from a cohort study of breast cancer survivors and showed that ginseng consumption was associated with an improved quality of life and survival.^4^ Results from these studies support the validity of ginseng intake data obtained in our study.

To minimize the bias from participants making changes in their ginseng consumption levels due to a disease diagnosis and preventing reverse causation, we excluded participants with less than 3 years of follow-up. Furthermore, although we adjusted for potential confounders, which were also self-reported, residual confounding remains possible, as some potential confounders could not be precisely ascertained. Lastly, the subjects in this study were female residents in Shanghai. Therefore, the results in the current study may not apply to males or other populations.

In conclusion, results from our study, along with support from recent findings, suggest potential health benefits of ginseng consumption in reducing mortality. Our study findings warrant further investigation on the potential health benefit of ginseng consumption.

## SUPPLEMENTARY MATERIAL

eTable 1. HRs^a^ and 95% CIs of mortality associated with short term and long term duration of ginseng use (years), Shanghai Women’s Health Study, 1996–2016.
