# Supplementary material for: Association of Ginseng Consumption With All-cause and Cause-specific Mortality: Shanghai Women’s Health Study
Source: J Epidemiol. 2022 Oct 5;32(10):469–75. doi: 10.2188/jea.JE20210393 (PMC9424189; doi:10.2188/jea.JE20210393)
Supplement: Supplementary file 1 [file je-32-469-s001.pdf]

eTable 1. HRs[a] and 95% CIs of mortality associated with short term and long term duration of ginseng use (years), Shanghai Women's Health Study, 1996-2016.								
	**Adjusted HR (95% CI)[a] among consumers for specific health benefits against existing illness[b]**				**Adjusted HR (95% CI)[a] among consumers for other reasons[c]**			
**Ginseng Consumption**	**Deaths (N)**	**< 3 years**	**Deaths (N)**	**≥ 3 years**	**Deaths (N)**	**< 3 years**	**Deaths (N)**	**≥ 3 years**
All causes								
Never	3,427	1.00	3,427	1.00	3,427	1.00	3,427	1.00
American/White ginseng	35	1.29 (0.90, 1.85)	103	1.22 (0.99, 1.49)	2	0.69 (0.18, 2.57)	5	0.99 (0.42, 2.30)
Cardiovascular diseases								
Never	1,049	1.00	1,049	1.00	1,049	1.00	1,049	1.00
American/White ginseng	7	0.72 (0.32, 1.62)	33	1.13 (0.79, 1.64)	2	2.98 (0.79, 11.20)	2	1.47 (0.38, 5.62)
Cancer								
Never	1,551	1.00	1,551	1.00	1,551	1.00	1,551	1.00
American/White ginseng	12	1.21 (0.68, 2.17)	30	0.97 (0.67, 1.40)	0	-	2	0.78 (0.19, 3.13)
Other disease causes								
Never	827	1.00	827	1.00	827	1.00	827	1.00
American/White ginseng	16	1.99 (1.20, 3.30)	40	1.57 (1.12, 2.19)	0	-	1	0.91 (0.13, 6.66)

BMI, body mass index, CI, confidence interval, HR, hazard ratio, WHR, waist-to-hip ratio.

[a] Adjustment: age, income, marriage status, education, menopause status, comorbidity (hypertension, diabetes), exercise, WHR, BMI, diet, vitamin and calcium supplements
